# Supplementary material for: Attached but Lonely: Emotional Intelligence as a Mediator and Moderator between Attachment Styles and Loneliness
Source: Int J Environ Res Public Health. 2022 Nov 11;19(22):14831. doi: 10.3390/ijerph192214831 (PMC9690062; doi:10.3390/ijerph192214831)
Supplement: Supplementary file 1 [file ijerph-19-14831-s001.zip › ijerph-2015467-supplementary.pdf]

Supplementary materials

Table S1. Alternative mediation analysis for Study 2 (with EI measurement excluding two TEIQue-SF items corresponding in terms of content to the Self-Worth subscale of the WAS).

Type	Effect	β	*SE*	95% CI LL	95% CI UL	*p*
Indirect	Avoidance ⇒ EI ⇒ Loneliness	0.187	0.016	0.043	0.106	< .001
	Avoidance ⇒ Self-worth ⇒ Loneliness	0.091	0.011	0.015	0.058	< .001
	Avoidance ⇒ Benevolence ⇒ Loneliness	0.012	0.005	−0.005	0.014	.330
	Anxiety ⇒ EI ⇒ Loneliness	0.072	0.013	0.011	0.061	.005
	Anxiety ⇒ Self-worth ⇒ Loneliness	0.078	0.013	0.013	0.064	.003
	Anxiety ⇒ Benevolence ⇒ Loneliness	−0.000	0.002	−0.004	0.004	.915
Component	Avoidance ⇒ EI	−0.508	0.039	−0.395	−0.244	< .001
	EI ⇒ Loneliness	−0.368	0.041	−0.313	−0.152	< .001
	Avoidance ⇒ Self-worth	−0.280	0.067	−0.403	−0.141	< .001
	Self-worth ⇒ Loneliness	−0.325	0.024	−0.180	−0.087	< .001
	Avoidance ⇒ Benevolence	−0.207	0.051	−0.243	−0.043	.005
	Benevolence ⇒ Loneliness	−0.056	0.031	−0.093	0.029	.299
	Anxiety ⇒ EI	−0.196	0.048	−0.248	−0.060	.001
	Anxiety ⇒ Self-worth	−0.239	0.084	−0.453	−0.126	< .001
	Anxiety ⇒ Benevolence	0.008	0.064	−0.118	0.132	.915
Direct	Avoidance ⇒ Loneliness	0.103	0.026	−0.011	0.093	.121
	Anxiety ⇒ Loneliness	0.158	0.029	0.023	0.135	.006
Total	Avoidance ⇒ Loneliness	0.368	0.027	0.103	0.210	< .001

Type	Effect	β	SE	95% CI LL	95% CI UL	p
	Anxiety ⇒ Loneliness	0.289	0.034	0.086	0.220	< .001

Table S2. Alternative mediation analysis for Study 1 (with participants' Gender[1] and Age as covariates).

Type	Effect	β	*SE*	95% CI *LL*	95% CI *UL*	*p*
Indirect	Avoidance ⇒ EI ⇒ Loneliness	0.22	0.04	0.14	0.30	< .001
	Anxiety ⇒ EI ⇒ Loneliness	0.16	0.04	0.09	0.24	< .001
Component	Avoidance ⇒ EI	-0.37	0.06	-0.50	-0.26	< .001
	EI ⇒ Loneliness	-0.59	0.03	-0.41	-0.29	< .001
	Anxiety ⇒ EI	-0.27	0.05	-0.32	-0.12	< .001
Direct	Gender ⇒ EI	0.09	0.16	-0.03	0.61	.075
	Age ⇒ EI	0.15	0.01	0.01	0.04	.005
	Avoidance ⇒ Loneliness	0.15	0.03	0.03	0.15	.006
	Anxiety ⇒ Loneliness	0.13	0.02	0.01	0.11	.012
	Gender ⇒ Loneliness	0.06	0.08	-0.04	0.27	.147
	Age ⇒ Loneliness	0.01	0.004	-0.01	0.01	.782
Total	Avoidance ⇒ Loneliness	0.37	0.04	0.15	0.30	< .001
	Anxiety ⇒ Loneliness	0.30	0.03	0.08	0.20	< .001

Note. Gender: Female (1), Male (2).

[1] In all analyses for Study 1 in which gender was controlled for, we did not include non-binary gender identity, as this category was represented by only two participants. However, we included non-binary participants in all other analyses reported in the text.

Table S3. Alternative mediation analysis for Study 2 (with participants' Gender and Age as covariates).

Type	Effect	β	*SE*	95% CI		*p*
				LL	*UL*	
Indirect	Avoidance ⇒ EI ⇒ Loneliness	0.18	0.05	0.08	0.29	< .001
	Avoidance ⇒ Self-worth ⇒ Loneliness	0.09	0.04	0.03	0.17	.013
	Avoidance ⇒ Benevolence ⇒ Loneliness	0.01	0.02	-0.01	0.05	.395
	Anxiety ⇒ EI ⇒ Loneliness	0.06	0.03	0.02	0.12	.022
	Anxiety ⇒ Self-worth ⇒ Loneliness	0.07	0.03	0.02	0.14	.011
	Anxiety ⇒ Benevolence ⇒ Loneliness	0.001	0.01	-0.01	0.01	.911
Component	Avoidance ⇒ EI	-0.51	0.04	-0.40	-0.25	< .001
	EI ⇒ Loneliness	-0.35	0.06	-0.35	-0.11	.002
	Avoidance ⇒ Self-worth	-0.29	0.06	-0.41	-0.16	< .001
	Self-worth ⇒ Loneliness	-0.31	0.04	-0.21	-0.07	.002
	Avoidance ⇒ Benevolence	-0.24	0.05	-0.27	-0.06	.002
	Benevolence ⇒ Loneliness	-0.06	0.03	-0.10	0.03	.295
	Anxiety ⇒ EI	-0.17	0.05	-0.24	-0.04	.005
	Anxiety ⇒ Self-worth	-0.23	0.08	-0.44	-0.12	.001
	Anxiety ⇒ Benevolence	- 0.01	0.06	-0.14	0.12	.878
	Gender ⇒ EI	0.07	0.13	-0.11	0.40	.271
	Gender ⇒ Self-worth	0.03	0.21	-0.33	0.52	.658
	Gender ⇒ Benevolence	-0.15	0.17	-0.67	0.002	.051
	Age ⇒ EI	0.13	0.01	0.003	0.04	.024
	Age ⇒ Self-worth	0.30	0.02	0.04	0.11	< .001
	Age ⇒ Benevolence	0.07	0.01	-0.01	0.04	.337
Direct	Avoidance ⇒ Loneliness	0.09	0.03	-0.01	0.09	.157
	Anxiety ⇒ Loneliness	0.15	0.03	0.02	0.14	.005
	Gender ⇒ Loneliness	0.01	0.07	-0.13	0.16	.817
	Age ⇒ Loneliness	0.06	0.01	-0.005	0.02	.235
Total	Avoidance ⇒ Loneliness	0.37	0.03	0.10	0.21	< .001
	Anxiety ⇒ Loneliness	0.29	0.03	0.08	0.22	< .001

Note. Gender: Female (1), Male (2).

Table S4. Alternative moderation analyses for Study 1 (with participants' Gender and Age as covariates).

				95% CI		
**Predictor**	β	***SE***	***t***	***LL***	***UL***	***p***
Intercept		0.19	8.90	1.35	2.12	< .001
Gender	0.05	0.08	1.17	-0.06	0.25	.59
Age	0.01	0.004	0.16	-0.01	0.01	.011
Avoidance	0.16	0.03	2.96	0.03	0.16	.089
Anxiety	0.10	0.03	1.79	-0.005	0.10	.075
EI	-0.60	0.03	-11.20	-0.41	-0.29	< .001
Anxiety x EI	-0.09	0.02	-1.98	-0.07	-0.0002	.049
Model summary				$R^2 = .59$<br>$F(6, 237) = 56.35, p < .001$		
Intercept		0.14	11.37	1.30	1.85	< .001
Gender	0.06	0.08	1.31	-0.05	0.26	.191
Age	0.01	0.004	0.27	-0.01	0.01	.784
Avoidance	0.14	0.03	2.62	0.02	0.15	.009
Anxiety	0.13	0.02	2.49	0.01	0.11	.013
EI	-0.60	0.03	-11.18	-0.41	-0.29	< .001
Avoidance x EI	-0.06	0.02	-1.48	-0.07	0.01	.140
Model summary				$R^2 = .58$<br>$F(6, 237) = 55.66, p < .001$		

Note. Gender: Female (1), Male (2).

Table S5. Alternative moderation analyses for Study 2 (with participants' Gender and Age as covariates).

				95% CI		
**Predictor**	β	***SE***	***t***	***LL***	***UL***	***p***
Intercept		0.19	8.90	1.35	2.12	< .001
Gender	0.03	0.08	0.53	-0.11	0.19	.593
Age	0.002	0.006	0.04	-0.01	0.01	.968
Avoidance	0.08	0.03	1.32	-0.02	0.09	.189
Anxiety	0.19	0.03	3.55	0.05	0.16	.001
EI	-0.61	0.04	-9.50	-0.49	-0.32	< .001
Anxiety x EI	-0.13	0.03	-2.40	-0.15	-0.01	.018
Model summary				$R^2 = .52$<br>$F(6, 179) = 32.95, p < .001$		
Intercept		0.21	7.26	1.10	1.92	< .001
Gender	0.03	0.08	0.53	-0.11	0.19	.593
Age	0.002	0.006	0.03	-0.01	0.01	.974
Avoidance	0.05	0.03	0.82	-0.03	0.08	.415
Anxiety	0.20	0.03	3.47	0.04	0.16	.001
EI	-0.61	0.04	-9.28	-0.49	-0.32	< .001
Avoidance x EI	-0.07	0.02	-1.21	-0.07	0.02	.227
Model summary				$R^2 = .51$<br>$F(6, 179) = 31.50, p < .001$		

Note. Gender: Female (1), Male (2).
